# Outpatient antibiotic prescribing for acute respiratory infections in Vietnamese primary care settings by the WHO AWaRe (Access, Watch and Reserve) classification: An analysis using routinely collected electronic prescription data

**DOI:** 10.1016/j.lanwpc.2022.100611

**Published:** 2022-10-11

**Authors:** Nam Vinh Nguyen, Nga Thi Thuy Do, Dung Tien Viet Vu, Rachel C. Greer, Sabine Dittrich, Maida Vandendorpe, Thach Ngoc Pham, Ngan Thi Dieu Ta, Thai Quang Pham, Vinh Thanh Khuong, Thuy Thi Bich Le, Lai Tuan Anh, Thai Hung Cao, Tung Son Trinh, Ha Thanh Nguyen, Long Nhat Ngo, Thom Thi Vu, H. Rogier van Doorn, Yoel Lubell, Sonia O. Lewycka

**Affiliations:** a Oxford University Clinical Research Unit, Hanoi, Vietnam; b Hanoi University of Pharmacy, Hanoi, Vietnam; c Mahidol Oxford Tropical Medicine Research Unit, Bangkok, Thailand; d Nuffield Department of Clinical Medicine, University of Oxford, Oxford, United Kingdom; e The Foundation of Innovative New Diagnostics, Genève, Switzerland; f National Hospital for Tropical Diseases, Hanoi, Vietnam; g National Institute of Hygiene and Epidemiology, Hanoi, Vietnam; h Nam Dinh Department of Health, Vietnam; i Nam Dinh Center for Diseases Control and Prevention, Vietnam; k Ministry of Health, Hanoi, Vietnam

**Keywords:** Antibiotics, Antimicrobial resistance, Primary care, Vietnam, Acute respiratory infections, WHO AWaRe

## Abstract

**Background:**

This study aims to investigate patterns of antibiotic prescribing and to determine patient-specific factors associated with the choice of antibiotics by the World Health Organization's Access-Watch-Reserve (WHO AWaRe) class for acute respiratory infections (ARIs) in rural primary care settings in northern Vietnam.

**Methods:**

We retrospectively reviewed health records for outpatients who were registered with the Vietnamese Health Insurance Scheme, visited one of 112 commune health centres in 6 rural districts of Nam Dinh province, Vietnam during 2019, and were diagnosed with ARIs. Patient-level prescription data were collected from the electronic patient databases. We used descriptive statistics to investigate patterns of antibiotic prescribing, with the primary outcomes including total antibiotic prescriptions and prescriptions by WHO AWaRe group. We identified patient-specific factors associated with watch-group antibiotic prescribing through multivariable logistic regression analysis.

**Findings:**

Among 193,010 outpatient visits for ARIs observed in this study, 187,144 (97.0%) resulted in an antibiotic prescription, of which 172,976 (92.5%) were access-antibiotics, 10,765 (5.6%) were watch-antibiotics, 3366 (1.8%) were not-recommended antibiotics. No patients were treated with reserve-antibiotics. The proportion of watch-antibiotic prescription was highest amongst children under 5-years old (18.1%, compared to 9.5% for 5–17-years, 4.9% for 18–49-years, 4.3% for 50–64-years, and 3.7% for 65-and-above-years). In multivariable logistic regression, children, district, ARI-type, comobid chronic respiratory illness, and follow-up visit were associated with higher likelihood of prescribing watch-group antibiotics.

**Interpretation:**

The alarmingly high proportion of antibiotic prescriptions for ARIs in primary care, and the frequent use of watch-antibiotics for children, heighten concerns around antibiotic overuse at the community level. Antimicrobial stewardship interventions and policy attention are needed in primary care settings to tackle the growing threat of antibiotic resistance.

**Funding:**

This work was supported through Australian government and UK aid from the UK government funding to FIND (Foundation for Innovative New Diagnostics) grant number FO17-0015, in addition to a Wellcome Trust grant (213920/Z/18/Z), and an Oxford University Clinical Research Unit internal grant from the Wellcome Trust Africa Asia Programme core grant in Vietnam (106680/Z/14/Z).

**Research in context**

**Evidence before this study**

We searched PubMed and Google Scholar for articles published from 1st January 2000 to 30th May 2021 using key concept terms such as “antibiotic” (“antimicrobial”, “antibacterial”), "primary care" ("community"), "prescribing" ("prescription", "use", "consumption"), "respiratory tract infection" ("respiratory infection") and "low middle income". We only considered the articles published in English.

We found that the previous studies on similar topics conducted in low-middle income countries mainly collected data from surveys or a sample of traditional handwritten prescriptions, which often contained a small sample size and lacked detailed and reliable clinical information on diagnoses and drug names, and possibly resulted in recall bias and documentation errors. Only a few studies have explored the factors associated with restricted antibiotic prescription, most of which used qualitative approaches that might limit representativeness, and they did not identify the most important determinants. Because the World Health Organization Access, Watch and Reserve antibiotic classification (WHO AWaRe) was published in 2017, the number of studies in low-middle income countries that have so far used it for data analysis remains limited.

**Added value of this study**

This is the first study in Vietnam to use routinely collected electronic health record data to characterize antibiotic prescribing for acute respiratory infections (ARIs) in primary care, and to determine which factors are associated with restricted antibiotic prescription by the World Health Organization Access, Watch and Reserve classification (WHO AWaRe). Electronic health record data are reliable because they are regularly checked and used as evidence of prescribers’ clinical practice at CHC for routine audits by local health authorities and the national reimbursement agency. Including nearly 200,000 outpatient visits from 112 commune health centres in Northern Vietnam during 2019, this study is the largest study in Vietnam on this topic to date making the findings robust. The findings of this study, including the patterns of antibiotic prescribing, and determinants of watch-antibiotic prescribing are critical to inform future policy-making on antibiotic prescribing and antibiotic stewardship in Vietnam, and as evidence to explain the alarming rise of antimicrobial resistance (AMR) in the country.

**Implications of all the available evidence**

Among 193,010 outpatient visits for ARIs observed in this study, 187,144 (97.0%) resulted in an antibiotic prescription, (172,976 [92.5%] were access-antibiotics, 10,765 [5.6%] were watch-antibiotics, 3,366 [1.8%] were non-recommended antibiotics), and no patients were treated with reserve antibiotics. Antibiotic prescription proportion was higher than 97% in the most commonly seen ARIs (acute pharyngitis [97.2%] and acute bronchitis [97.3%]) and higher than 90% in ten of eleven types of ARI observed. Influenza, with the lowest antibiotic prescription proportion, still reached 77.3%. To our knowledge, it is the highest all-age proportion of antibiotic prescribing for common infections which has been observed in Vietnamese primary care settings. This proportion is much higher than those reported from High Income Countries’ health systems as well as other AMR hotspots. Of concern, our study also found that the proportion of watch-antibiotic prescribing in children was higher than that in adults, especially for those under 5-year old (18.1%, compared with 4.9% in adults aged 18–49-year old), reflected by both descriptive data and regression analyses. This pattern is in line with what has been observed in other primary care settings in Vietnam, such as community pharmacies and outpatient clinics at urban hospitals, underscoring that children should be an important target for antibiotic stewardship interventions. To address these situations, we believe that strengthening primary healthcare prescribers’ capacity through interventions such as education, training in communication skills, developing appropriate guidelines, and providing them with point-of-care diagnostic tests to guide antibiotic prescribing is crucial.

Alt-text: Unlabelled box

## Introduction

Acute respiratory infections (ARIs) are the most frequent infections presenting to primary care in low- and middle- income countries (LMICs).^1^, ^2^, ^3^ ARIs are also the most common reason for antibiotic prescription in both adults and children, despite a predominantly viral aetiology.^4^, ^5^, ^6^ Even for ARIs commonly associated with a bacterial aetiology such as sinusitis and otitis media, evidence-based guidelines do not recommend immediate antibiotic prescription.^7^^,^^8^ Excessive use of antibiotics for common ARIs puts patients at risk for antibiotic-related adverse reactions and facilitates the development of antimicrobial resistance (AMR) at both patient and societal levels.

As the world is exiting the fast and intense pandemic of COVID-19, a slower and more insidious pandemic of AMR is gradually worsening.^9^^,^^10^ According to the most comprehensive estimate on the global burden of bacterial AMR to date, it was associated with 4.95 million deaths and directly resulted in 1.27 million deaths worldwide in 2019, with LMICs shouldering the higher burden.^11^ Vietnam, which is both a hotspot for emerging infections and for AMR, has seen an alarming reduction in susceptibility of pneumococci to different antibiotics.^12^ Between 1999 and 2007, for instance, the proportion of *Streptococcus pneumoniae* with reduced susceptibility to penicillin increased from 8% to 75%.^13^ A recent study noted that susceptibility of *S.pneumoniae* and *Haemophilus influenza* was only 3% for macrolides and 5% for 1st and 2nd generation cephalosporins, consequently ranking the antibiotic susceptibility in Vietnam among the lowest in Asia.^14^ This disturbing rise of AMR in Vietnam is driven by both over-prescription of antibiotics at different levels of healthcare as well as unrestricted access and use of antibiotics in community pharmacies.^15^^,^^16^

The current health system in Vietnam is a mixed public-private system, in which the public system plays a critical role in preventive and curative care for the population nationwide, and almost 90% of the population have national health insurance.^17^ Commune health centres (CHCs) are the foundation of public primary care, including a network of more than 11,000 centres that provide basic and essential health services in every Vietnamese commune. A CHC is usually staffed with a general doctor and some ancillary staff such as a midwife, nurse or pharmacist. Among them, only general doctors (or assistant doctors in remote communes) are legally allowed to prescribe antibiotics.^18^^,^^19^ To date, Vietnam has not released specific treatment guidelines for the management of infectious diseases or antibiotic treatment in primary care settings.

There are concerns that over-prescription of antibiotics for ARI symptoms in CHCs in Vietnam has contributed to the emergence and spread of AMR. A large survey in 217 CHCs in Hai Phong Province from 1994 to 1996 showed that over 51% of patients visiting CHCs were for ARIs and 65% were prescribed antibiotics.^20^ A more recent study reviewed a random sample of 1047 out of 41,847 available prescriptions from 11 CHCs in one district (My Loc) in Nam Dinh Province found that the proportion of antibiotic prescriptions for ARIs was around 90% depending on the type of ARI.^21^

These previous studies in Vietnam mainly collected data from surveys or a sample of traditional handwritten prescriptions, which often lacked detailed and reliable clinical information on diagnoses and drug names, and possibly resulted in recall bias and documentation errors. Since the release of the Vietnamese National Action Plan on AMR in 2013, large-scale studies updating evidence on the context of antibiotic treatment in primary care settings in Vietnam has been limited. Therefore, we conducted this study to provide updated data on antibiotic prescribing by general practitioners in rural primary healthcare centres in northern Vietnam and to identify patient-specific factors associated with choice of antibiotics.

## Methods

### Study design and settings

We conducted a retrospective review of claims data from Electronic Health Records (EHRs) for outpatients at 112 CHCs in six rural districts (Truc Ninh, Nam Truc, Y Yen, Hai Hau, Xuan Truong and Nghia Hung) in Nam Dinh province, northern Vietnam. Profiles of these districts are provided in Supplementary Table 1. Nam Dinh is 90 km southeast of Hanoi, the capital city. The province covers an area of 1,669 km2 and supports a population of approximately 1.781 million (population density: 1067 persons per km^2^). It includes 1 city and 9 rural districts with 194 rural communes. The monthly average income per capita of residents in 2021 in Nam Dinh province (4.615 million VND) was a bit higher than that in the whole country (4.30 million VND).^22^

The CHCs included in this study were the study sites of two cluster-randomised trials aiming to investigate the effectiveness of interventions to improve antibiotic use in the community, which were conducted in six rural districts in Nam Dinh province. The first trial, assessing the effectiveness of C-reactive protein point-of-care testing (ICAT) to guide antibiotic prescribing for ARIs, included 48 CHCs in three districts (Truc Ninh, Nam Truc, and Y Yen).^23^ The second trial, assessing the impacts of educational and collective action interventions delivered in primary healthcare (Co-Act), included 64 CHCs in three other districts (Hai Hau, Xuan Truong, and Nghia Hung). Both trials selected CHCs by the following eligibility criteria: (1) serving a commune population larger than 3000 individuals; (2) having a licensed prescriber; and (3) having an electronic database for reporting patient level data. In these 112 CHCs, empiric treatment for infectious diseases is the norm, as diagnostic laboratories and tests supporting infection diagnosis are unavailable. Data for our study were collected in the year preceding the interventions, therefore prescribing behaviours of primary care doctors were not affected by the trial interventions, nor by COVID-19.

### Data sources and study participants

From the claims databases of 112 selected CHCs in this study, our study collaborators who were staff at district health centres extracted electronic health record (EHR) data (raw data) for all patients registered with the Vietnamese Health Insurance Scheme and who had at least one health visit at any of these centres between 1^st^ January and 31^st^ December 2019. Afterwards, these data were gathered and transferred to study coordinators (HTN and TTV). The coordinators checked for missing or potentially invalid data (e.g.. data on a visit was available but data on treatment was missing) and gave feedback to the study collaborators. The study collaborators discussed with CHC prescribers and re-checked the CHC's electronic database, or compared electronic data and data from the CHC's health record notebook to validate these cases and provided adjustments if needed. Finally, raw data were sent to our data manager (DTVV) to integrate and convert into a cleaned, validated and ready-to-use format. The data manager also removed patients’ and prescribers’ names to ensure confidentiality before analysis.

The EHRs include health insurance code, demographic data (age, sex, occupation, and village), clinical diagnosis, clinical symptoms, the international classification of diseases - 10th revision (ICD-10) code assigned for the main diagnosis, medications prescribed, and the location of the CHC.

We obtained EHRs of all outpatients who visited for ARIs, defined by an ICD-10 code relating to ARIs (H65-67, J00-06, J09-18, and J20-22), or a clinical diagnosis with ARI. We chose these codes and diagnoses by reviewing previously published research on a similar topic.^4^^,^^24^^,^^25^ These codes and diagnoses were confirmed by an infectious disease specialist (NTDT) in the study team. To avoid computerization errors, we validated these visits by examining whether the patient had at least one ARI-related symptom (e.g. cough, runny nose, sore throat and dyspnoea, pain inside the ear, fluid draining from ear [otitis media]), or was prescribed with at least one pharmacological treatment for respiratory illnesses (e.g. antibiotics, antihistamine, cough suppressants, short-term corticosteroids, mucolytic, or herbal medicines for respiratory illnesses). Our selection procedure initiated from prescribers' diagnoses, not ARI-related symptoms, because symptoms were inconsistently reported and could be seen in other conditions such as chronic respiratory or cardiovascular diseases and could cause misclassification.

### Antibiotic prescriptions

In this study, we defined an antibiotic prescription for ARI as related to a visit if at least one systemic antibiotic was prescribed. For the ARI visits with multiple diagnoses relating to infectious diseases, a study pharmacist (NVN) reviewed the relevant record to determine if antibiotic prescription targeted the ARI or the other diagnoses. An infectious disease specialist (NTDT) in the study team participated in the assessment process if the study pharmacist needed further consultation.

We presented data on patterns of antibiotic prescribing according to the 2021 Access-Watch-Reserve (AWaRe) antibiotic classification of the World Health Organization (WHO). According to this classification, 258 common antibiotics were classified into 4 groups: access, watch, reserve and not recommended. Access-group antibiotics are the first or second choice antibiotics that offer the best therapeutic value while minimizing the potential for resistance. Watch-group antibiotics are the first and second choice antibiotics for a specific, limited number of infective syndromes because these agents are more prone to development of antibiotic resistance and thus prioritized as targets of stewardship programs and monitoring. Finally, reserve-group antibiotics should be considered antibiotics of last resort, which should be tailored to highly selected patients (life-threatening infections due to multi-drug resistant bacteria) when all alternatives have failed or are not suitable. The classification also lists those antibiotics whose use is not recommended by WHO, namely fixed-dose combinations of multiple broad-spectrum antibiotics that lack evidence-based indications for use or recommendations in high-quality international guidelines. For visits with a combination of antibiotics, our classification was based on the antibiotic with the highest level of restriction (e.g. if visits contained both watch- and access- antibiotics, they were classified as watch-antibiotic encounters).

### Statistical analysis

Descriptive statistics were used to investigate the patterns of antibiotic prescribing for ARIs. The primary descriptive analysis includes the antibiotic prescription proportions for outpatient visits for ARIs, defined by the number of visits with antibiotic prescriptions divided by the total number of visits in selected CHCs during the studied duration. These proportions were measured for total antibiotics and for each AWaRe antibiotic category. We also calculated the prescription proportions of total antibiotics and of AWaRe-specific groups for each ARI, and for each sub-population stratified by age or by selected districts.

We analysed determinants of prescribing restricted antibiotics (either watch- or reserve-antibiotics). These agents have higher toxicity and/or resistance potential compared with access antibiotics and are not recommended as first choices for common infections such as community-acquired ARIs. Since reserve antibiotics are not available at primary care level in Vietnam, and were not found in our study population, our analysis only reviewed the determinants associated with prescribing watch-antibiotics. We performed a mixed effects logistic regression model to estimate odd ratios (ORs) and 95% CIs for the outcome of being prescribed at least one watch-group antibiotic for ARI. The variables included in the model were identified through a literature review of previously published studies with similar topics.^4^^,^^6^^,^^26^ Fixed effects included age (stratified into 5 groups comparable to a previous study), gender, district, clinical ARI diagnosis, and presence of comorbid chronic respiratory illness.^4^ CHC site and patient ID were included as random effects to take into account clustering effects by CHC and within patients with repeated visits, respectively. The ORs were converted into relative risks (RRs) for ease of interpretation.^3^^,^^27^^,^^28^ R version 3.3.3 was used for the statistical analysis.

### Ethical considerations

The protocol of the ICAT trial was approved by the Oxford University Tropical Research Ethics Committee (OxTREC, Reference number: 53–18) and the ethical committee of the National Hospital for Tropical Diseases in Vietnam (Reference: 07/HDDD-NDTW/2019). The protocol of the Co-Act trial (assessing the impacts of educational and collective action interventions delivered in primary healthcare on reducing prescription of antibiotics for patients with ARIs in routine care) was approved by the Oxford University Tropical Research Ethics Committee (OxTREC, Reference number: 528-19) and the ethical committee of the National Institute of Hygiene and Epidemiology (Reference: HDDD-30/2019). Permission to carry out this study was obtained from local authorities.

## Results

### Demographic characteristics

Among 409,139 outpatient visits at 112 CHCs in 6 districts in rural Nam Dinh in 2019, we identified 193,010 visits for ARIs (Table 1 and Supplementary Table 2). Patients’ median age was 57 (interquartile range 37–68) years. Female patients accounted for 103,823 (53.7 %) of the total visits. The number of visits in each district ranged from 19,447 (10.1% of the total visits) to 52,161 (27.0%). Acute pharyngitis (127,322 visits [66.0%]) and acute bronchitis (35,619 visits [18.5%]) accounted for the highest proportion of ARIs. In general, upper ARIs were more common than lower ARIs.

**Table 1:** Baseline characteristics of the outpatient visits diagnosed with ARIs in 112 CHCs in rural Nam Dinh in 2019.

	Number of health visits for ARIs (n=193,010)	
**Patient characteristics**		
Age, median (IQR) (years)	57	(37–68)
Age groups, n (%)		
<5	9185	(4.8)
5 to 17	29,453	(15.3)
18 to 49	36,749	(19.0)
50 to 64	50,949	(26.4)
≥ 65	66,674	(34.5)
Female gender, n (%)	103,722	(53.7)
Community (district), n (%)		
Truc Ninh	19,420	(10.1)
Nam Truc	28,380	(14.7)
Nghia Hung	34,978	(18.1)
Xuan Truong	21,859	(14.7)
Y Yen	52,139	(27.0)
Hai Hau	36,234	(18.7)
Acute respiratory infection diagnosis, n (%)		
[H65] Acute otitis media	1962	(1.0)
[J00] Acute nasopharyngitis [common cold]	7091	(3.7)
[J01] Acute sinusitis	3046	(1.6)
[J02] Acute pharyngitis	127,322	(66.0)
[J03] Acute tonsillitis	6721	(3.5)
[J04] Acute laryngitis and tracheitis	959	(0.5)
[J06] Acute upper respiratory infections of multiple/ unspecified sites	3436	(1.8)
[J09-J11] Influenza	2251	(1.2)
[J12-J18] Pneumonia	4524	(2.3)
[J20-J21] Acute bronchitis/bionchiolitis	35,619	(18.5)
[J22] Unspecified acute lower respiratory infection	79	(0.0)
Comorbid chronic respiratory illnesses, n (%)	6142	(3.2)
Child population (aged < 18-years)	347	(0.9)
Adult population (aged ≥ 18-years)	5795	(3.8)

Prevalence of child patients with comorbid chronic respiratory illnesses was calculated among child population (*n*=38,638).

Prevalence of adult patients with comorbid chronic respiratory illnesses was calculated among adult population (*n*=154,372).

### Patterns of antibiotic prescribing

**Figure 1:**
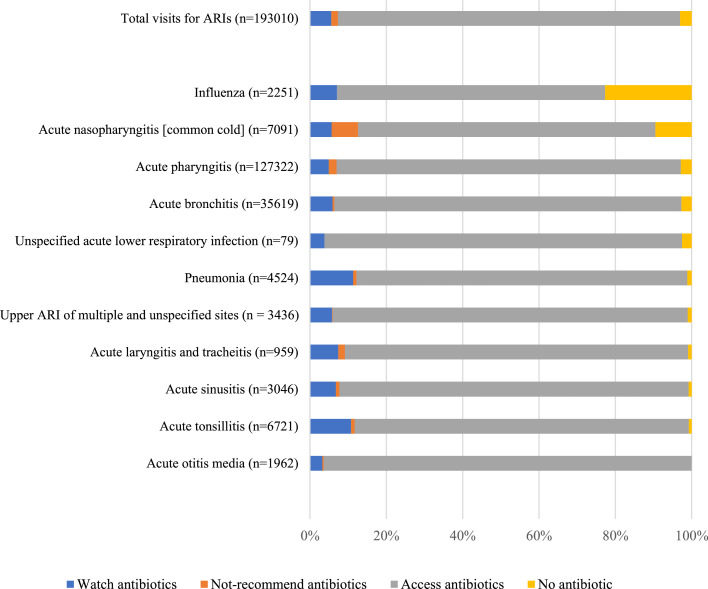
Antibiotic prescription proportion by ICD-10 diagnosis among health visits for ARIs to 112 CHCs in Nam Dinh province in 2019.

Among 193,010 ARI visits, 187,144 (97.0%) were prescribed at least one systemic antibiotic. Antibiotic prescription proportion was higher than 97% in the most commonly seen ARIs (acute pharyngitis [97.2%] and acute bronchitis [97.3%]) and higher than 90% in ten of eleven types of ARI observed. Even in patients diagnosed with influenza, by definition of viral aetiology, the proportion of patients prescribed an antibiotic reached 77.3%. Between WHO-AWaRe antibiotic groups, access-group antibiotics were the most frequently prescribed (172,976 visits [89.6% of the total visits for ARIs]). Watch-group and not-recommended antibiotics were less frequently prescribed, at 5.9% and 1.8%, respectively. No reserve-group antibiotics were prescribed. Prescriptions of watch-group antibiotics was highest in pneumonia and acute tonsillitis, at 11.3% and 10.0 %, respectively, and prescription of not-recommended antibiotics was highest in acute nasopharyngitis, at 7.7% (Figure 1, see also Supplementary document 3).

**Figure 2:**
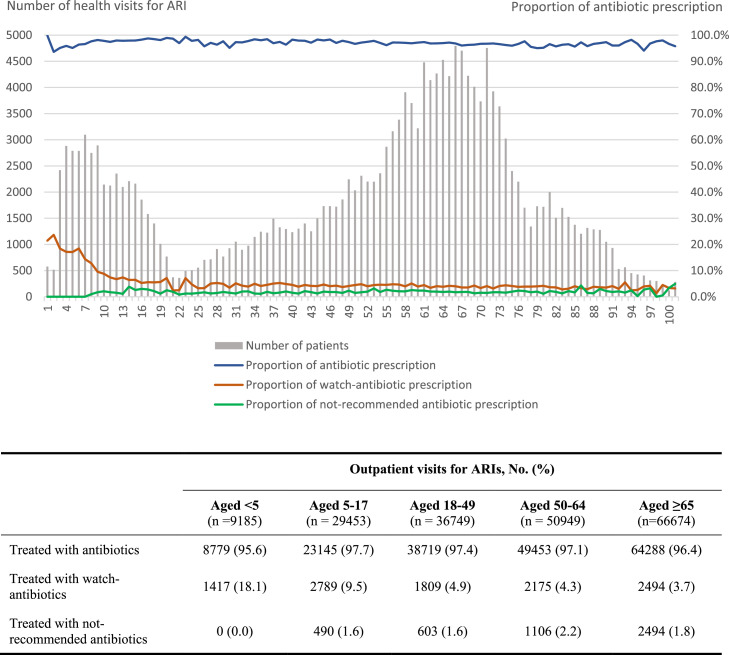
Antibiotic prescription proportions by age for health visits with ARIs at 112 CHCs in Nam Dinh province in 2019.

In all age groups, antibiotic prescription proportion was higher than 95%. Patients at younger ages, especially under 5-years, were prescribed watch-group antibiotics more frequently (0–4-years [18.1%], 5–17-years [9.5%], 18–49-years [4.9%], 50–64 [4.3%] and 65-and-above-years [3.7%]). Proportions of not-recommended antibiotic prescribing for all age groups were below 5% (Figure 2). Patients aged 0-4-years were not prescribed not-recommended antibiotics.

**Figure 3:**
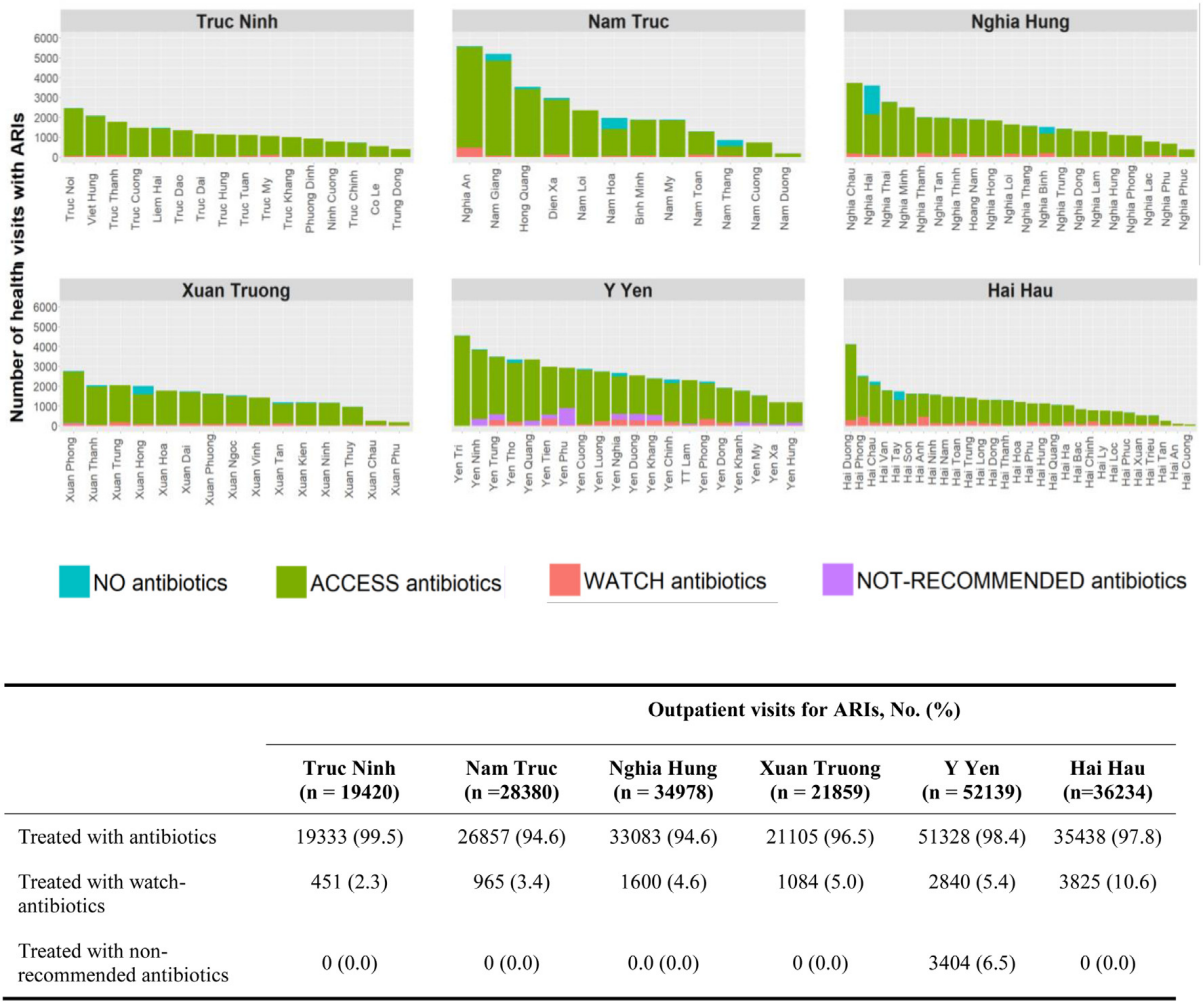
Antibiotic prescription proportions by geographic area for health visits with ARIs at 112 CHCs in Nam Dinh province in 2019.

In all studied districts, antibiotic prescription proportions were higher than 90%. Watch-group antibiotic prescription proportions were lower than 10% in all districts except for Hai Hau (10.6%). Y Yen was the only district where not-recommended antibiotics were prescribed (Figure 3).

### Factors associated with prescribing watch-antibiotics

**Figure 4:**
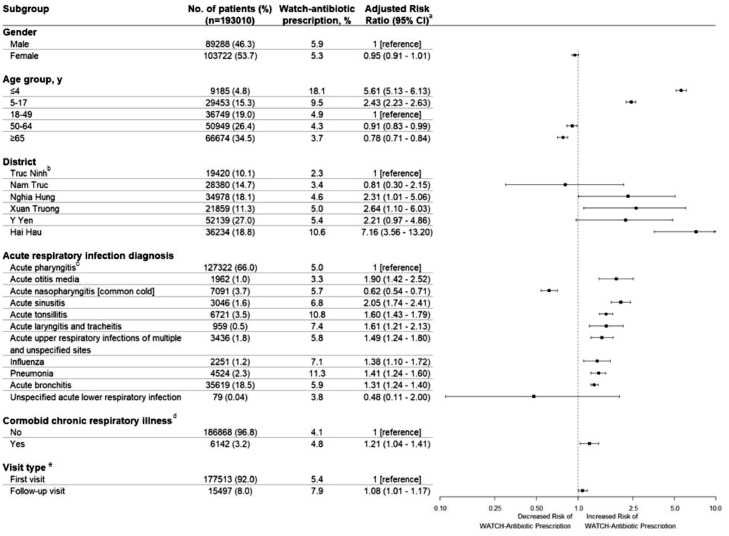
Factors associated with watch-antibiotic prescription for patients with ARIs. a. A mixed effects logistic regression model with ORs converted to RRs. Fixed effects included age, gender, district, clinical ARI diagnosis, presence of comorbid chronic respiratory illness, and visit type. Random effects included commune health centers and patient ID. b. Truc Ninh was chosen as reference because of the lowest watch-antibiotic prescribing proportion and no other normative group. c. Acute pharyngitis was chosen as reference because it was the most commonly seen ARI. d. No comorbid illness was the normative category and used as reference. e. the first visit if there was no visit for ARI in the same patient within the prior 30 days (normative reference group), and second visit if there was at least one visit for ARI in the same patient within the prior 30 days.

We found that younger age, district, type of ARI, presence of comorbid chronic respiratory illness, and follow-up visits were associated with prescribing watch-antibiotics. Compared with adults aged 18–49-years, children aged under 5-years (aRR, 5.61; 95%CI, 5.13 to 6.13), and those aged 5–17-years (aRR, 2.43; 95%CI, 2.23 to 2.63) had more risk to be prescribed watch-antibiotics, while older adults aged 50–64-years (aRR, 0.91; 95%CI, 0.83 to 0.99) and the elderly aged 65-and-above-years (aRR, 0.78; 95%CI, 0.71 to 0.84) were less likely to be prescribed watch-antibiotics. Between districts, Nghia Hung, Xuan Truong and Hai Hau had higher watch-antibiotic prescribing compared with Truc Ninh. Most of the less common ARIs had higher watch-antibiotic prescription compared with acute pharyngitis (the most commonly seen ARI), with the exception of acute nasopharyngitis (lower prescribing) and unspecified lower respiratory tract infection. Watch-antibiotics were more likely prescribed in follow-up visits rather than first visits (aRR, 1.08; 95%CI, 1.01 to 1.17). (Figure 4) Patients with comorbid chronic respiratory illnesses had higher risk than those without these illnesses (aRR, 1.21; 95%CI, 1.04 to 1.41), but our sub-group analyses found that this factor significantly increased the risk only in adult patients (aRR, 1.19; 95%CI, 1.03 to 1.38), not in child patients (aRR, 0.86; 95%CI, 0.58 to 1.26) (Figure 4 and Supplementary Document 4).

## Discussion

In this study, 97% of the total visits for ARIs received antibiotic prescriptions. To our knowledge, it is the highest all-age proportion of antibiotic prescribing for common infections which has been observed in Vietnamese primary care settings. This proportion eclipses those reported in high income countries such as in the US (41%), the UK (7.5–30.9% in) and Japan (52.7%), as well as other AMR hotspots in India (45.1%), China (52.9%) and South America (35–40%).^4^^,^^6^^,^^29^, ^30^, ^31^, ^32^ Compared with other South East Asian countries, the proportion of antibiotic prescribing for ARIs in Vietnamese primary care was much higher than in Malaysia (49.2%) and in Thailand (10–88% depending on the diagnosis), but quite similar to that in Myanmar (96%).^33^, ^34^, ^35^ Even for ARIs that by definition do not require antibiotics such as influenza, the proportion of antibiotic prescribing in Vietnam remains alarmingly high (77.3% vs. 10% in the US and 19% in Japan).^4^^,^^36^ It should be noticed that our database included only information on antibiotic prescriptions at CHCs. This means that patients diagnosed with severe ARIs (e.g. complicated pneumonia) at CHCs, indicating the need for referral to higher-level healthcare settings (i.e. district or provincial hospital), could be treated with antibiotics at higher-level healthcare settings instead of CHCs. Therefore, the overall proportion of antibiotic treatment in our study population in real-life could be even higher than what was observed in this study (97%).

The prescription proportion of access-antibiotics was much higher than watch- antibiotics (92.5% vs 5.6%, respectively), which is in line with the WHO recommendations on prioritizing access-antibiotics as first-line treatment for community-acquired infections. This highlights the success of tightening prescribing policies in improving appropriate use of antibiotics.^37^ In Vietnam, the national reimbursement program limits its payment for watch-antibiotics and does not cover reserve antibiotics at primary healthcare level. Specifically, the national reimbursement drug list for CHCs only includes cefuroxime among advanced-generation cephalosporins, only erythormycin and spiramycin among macrolides, and only ciprofloxacin and ofloxacin among oral fluoroquinolones.^38^ To minimize the overuse of fluoroquinolones, Vietnamese health authorities have also categorized these agents in the list of medications requiring rigorous monitoring since 2017.^39^ These interventions help preserve watch-antibiotics as second-line treatment for common infections and prevent massive prescription of these agents, as observed at other AMR hotspots such as India where primary care doctors prescribed macrolides and fluoroquinolones for nearly 45% of patients with uncomplicated ARIs.^30^

Of concern, our study found that primary care doctors in rural Vietnam use more watch-antibiotics for children than for adults, especially for those under 5-year old, reflected by both descriptive data and regression analyses. Watch-antibiotics prescribed for children in our study population included broad-spectrum antibiotics such as second generation cephalosporins and macrolides, which were previously shown not to improve clinical outcomes in children with ARI compared with narrow-spectrum antibiotics, while increasing the risks of drug-related adverse events.^40^^,^^41^ Common use of watch-antibiotics in children was also found at community pharmacies in rural Vietnam, where the proportion of watch-antibiotic dispensing for children was nearly 55% and was 1.5 times higher than that for adults.^42^ Similarly, a study conducted at the outpatient clinic of an urban hospital in Vietnam found that watch-antibiotics including cefuroxime (22.0% of the total prescriptions), cefixime (11.4%), cefaclor (8.2%), erythromycin (3.7%) and cefpodoxim (2.3%) were the most commonly prescribed for mild respiratory illnesses. Although the mechanisms resulting in this pattern need to be further investigated, the high use of watch-antibiotics for children with mild illnesses in Vietnam, which was observed consistently across different primary care settings, underscores that children should be an important target for antibiotic stewardship interventions. Overuse of these agents in the early stages of life is associated with decreasing efficacy of antibiotic treatment and driving antimicrobial resistance in the future.^43^

We also observed different patterns of antibiotic prescribing by district. In this study, Hai Hau district, the one with the highest population and number of health facilities, also had the highest proportion of watch-antibiotic prescribing.^22^ Y Yen district, with the second highest population and number of health facilities, also had the second highest proportion of watch-antibiotic prescribing, and was the only district dispensing not-recommended antibiotics, i.e. amoxicillin and cloxacillin, the agent that was considered to be similar to amoxicillin and clavulanic acid by some prescribers. In these larger districts, the centralized purchasing unit can use its population size and heterogeneous requests from different health settings in their catchment area as arguments to demand more restricted antibiotics.^44^ Therefore, to manage the use of restricted antibiotics at primary-care level, antibiotic stewardship interventions should target not only the prescribers at commune level, but also the antibiotic selection processes at district-level.

Vietnam was one of the first countries to develop a National Action Plan to combat AMR in the World Health Organization (WHO) Western Pacific Region. This plan addresses raising awareness of AMR within the community, safeguarding access to antimicrobials and encouraging the safe use of drugs as among key ways to prevent the spread of AMR. However, prescribers’ habits and diagnostic uncertainty contribute to antibiotic misuse.^45^ Despite local and international efforts to educate patients and enforce prescribing policies, our study raises concerns that antibiotic misuse seems to be getting worse in Vietnam. To address this situation, we believe that strengthening primary healthcare prescribers’ capacity through interventions such as education, training in communication skills, developing appropriate guidelines, and providing them with point-of-care diagnostic tests to guide antibiotic prescribing is crucial.^46^ A new WHO Essential Medicines List Antibiotic Book will be released later this year. Translating this into local guidelines and training doctors in how to follow them will be an important effort in antimicrobial stewardship in primary care settings. Patients’ demand for antibiotics is also cited as a key driver for antibiotic over-consumption.^47^ Previous evidence reveals that this pressure sways prescribers towards prescribing unnecessary antibiotics.^48^ It is worth remembering that CHCs are not the only source of antibiotics for ARI patients in the community. Similar to other LMICs, antibiotic self-medication through retail pharmacies in Vietnam is very common, and 90% of antibiotics may be dispensed without a valid prescription.^15^ The proportion of watch-group antibiotics bought over-the-counter in pharmacies is much higher than at CHCs, and needs to be more tightly regulated.^42^ High availability and the ease of access to antibiotics by the general public was confirmed as a fundamental driver of antibiotic resistance.^49^ A recent study shows that antibiotics were inappropriately supplied to 92% of adults requesting treatment for upper respiratory tract infection symptoms at community pharmacies.^50^ Because a course of antibiotic treatment is generally not costly, patients will purchase antibiotics at retail pharmacies if a prescriber refuses to prescribe antibiotics without solid evidence that antibiotic treatment is unnecessary.^51^

To our knowledge, this is the first study in Vietnam to use routinely collected EHR data to characterize antibiotic prescribing for ARIs in primary care, and to determine which factors are associated with restricted antibiotic prescription. Including outpatients from 112 CHCs, this study is the largest study on this topic to date (nearly 200,000 health visits) making the findings robust. This also minimizes impacts of recall and responder bias because data was immediately collected at the time of patient visits and documented in CHCs’ electronic database. Electronic health record data are reliable because they are regularly checked and used as evidence of prescribers’ clinical practice at CHC for routine audits by local health authorities and the national reimbursement agency.^52^ The combined data on antibiotic prescription with patient-level factors allows exploration of determinants of antibiotic prescribing. However, this study has several limitations. Firstly, outpatient claims data lacks detailed clinical information including previous antibiotic treatment, allergy history, and laboratory testing results. Therefore, it was difficult to differentiate between appropriate and inappropriate antibiotic prescriptions. We also could not investigate association between prescriber's characteristics and antibiotic choice because data on prescriber's name and speciality were frequently lacking (only found in around 60% of the EHRs) and inaccurate (the staff assigned for inputting data in several CHCs input their name instead of prescriber's name in the record). Secondly, our study sites are rural, government facilities located in one region (Nam Dinh province), which limits representativeness of our findings for other settings (urban, private primary care, other regions of Vietnam). However, our results might be generalisable to other rural regions because the studied CHCs were distributed across all types of rural regions (mid-size towns, small towns and villages), and the average income per month of residents in our regions was quite similar to that of Vietnamese residents (ranged from 4.37 to 4.42 million VND, compared with 4.2 million VND [2021]). (Supplementary document 1). Thirdly, our findings are limited to patients with national health insurance, who might be treated and receive medications differently to those who use private primary care services. Almost 90% of the patient population at CHCs have health insurance, so this is unlikely to have a large effect on the overall results.^17^ Lastly, since we used available claims data, we cannot confirm all patients are valid. During our visits to CHCs to assess the feasibility of this study, prescribers revealed that some patients might use their health insurance ID to receive medications for family members not participating in the national insurance program. Therefore, there may be some misclassification of patient details, but the prescribing behaviours and antibiotics supplied should be of good accuracy.

In conclusion, our study finds an alarmingly high proportion of antibiotic prescription for ARIs in public primary care settings in Vietnam. These findings corroborate earlier reports regarding inappropriate antibiotic prescribing in rural Vietnam, and other LMICs. There is an urgent need for multi-faceted interventions to tackle both over-prescribing and choice of antibiotics. These could include training in antimicrobial stewardship and communication skills, and point-of-care diagnostic tests to guide antibiotic treatment decisions.

## Contributors

NVN, NTTD, NTDT, RCG, SD, MV, HRvD, YL and SOL were responsible for conceptualization and study design. HTN and TTV coordinated study activities across the sites with supervision from NTTD and SOL, and local support and oversight by TNP, NTDT, TQP, VTK, TTBL and THC. DTVV and TST accessed, cleaned, managed and verified the data. NVN led the data analysis with supervision from DTVV and SOL. LNN supported the data analysis and visualization. NVN drafted the manuscript with supervision from SOL. All authors contributed to the final version of the manuscript and approved the submission. The corresponding author had final responsibility to submit for publication.

## Data sharing statement

Data may be available according to data sharing policy of the relevant partners contributing to this study upon request to the corresponding author.

## Declaration of interests

The authors declared no conflicts of interest.

## Appendix Supplementary materials

Image, application 1

Image, application 2

Image, application 3

Image, application 4

Image, application 5
