# Supplementary material for: Outpatient antibiotic prescribing for acute respiratory infections in Vietnamese primary care settings by the WHO AWaRe (Access, Watch and Reserve) classification: An analysis using routinely collected electronic prescription data
Source: Lancet Reg Health West Pac. 2022 Oct 11;30:100611. doi: 10.1016/j.lanwpc.2022.100611 (PMC9677071; doi:10.1016/j.lanwpc.2022.100611)
Supplement: Supplementary file 1 [file mmc1.docx]

**SUPPLEMENTARY DOCUMENT 3**

Comparison by districts towards population size, average income and health facility

|  | **Truc Ninh** | **Nam Truc** | **Nghia Hung** | **Xuan Truong** | **Y Yen** | **Hai Hau** |
| --- | --- | --- | --- | --- | --- | --- |
| Population size | 172932 | 184061 | 176225 | 150899 | 228766 | 262244 |
| Population at rural area | 140424 | 166305 | 154760 | 142458 | 218207 | 237527 |
| Average income (millions VND/month/person) | 4,392 | 4,371 | 4,405 | 4,327 | 4,318 | 4,415 |
| Total number of primary healthcare settings | 20 | 20 | 24 | 21 | 31 | 34 |
| Total number of beds of primary health settings | 95 | 100 | 255 | 100 | 214 | 185 |
| Proportion of communes having medical doctor by district (%) | 100.0 | 65.0 | 100.0 | 90.0 | 75.0 | 80.0 |
