# Supplementary material for: Outpatient antibiotic prescribing for acute respiratory infections in Vietnamese primary care settings by the WHO AWaRe (Access, Watch and Reserve) classification: An analysis using routinely collected electronic prescription data
Source: Lancet Reg Health West Pac. 2022 Oct 11;30:100611. doi: 10.1016/j.lanwpc.2022.100611 (PMC9677071; doi:10.1016/j.lanwpc.2022.100611)
Supplement: Supplementary file 2 [file mmc2.docx]

**SUPPLEMENTARY DOCUMENT 1**

**Diagram to select acute respiratory infection patients into the study**

Total number of patient visits to 112 commune health centers (CHCs) in 6 rural districts in Nam Dinh province from 1^st^ January to 31^st^ December 2019

N = 409139

Total number of visits with acute respiratory infection (ARI) diagnosis according to the International Classification of Diseases – 10^th^ version (defined either by ICD-10 code or related diagnosis)

N = 193170

Total number of visits with ARI diagnosis, validated by at least one ARI symptoms (e.g. cough, runny nose, sore throat, dyspnoea, pain inside the ear, fluid draining from ear [otitis media]) OR pharmacological treatment for respiratory illnesses (e.g. antibiotics, antihistamine, cough suppressants, short-term corticosteroids, mucolytic and herbal medicines for respiratory illnesses)

N = 193010
