# Supplementary material for: Outpatient antibiotic prescribing for acute respiratory infections in Vietnamese primary care settings by the WHO AWaRe (Access, Watch and Reserve) classification: An analysis using routinely collected electronic prescription data
Source: Lancet Reg Health West Pac. 2022 Oct 11;30:100611. doi: 10.1016/j.lanwpc.2022.100611 (PMC9677071; doi:10.1016/j.lanwpc.2022.100611)
Supplement: Supplementary file 3 [file mmc3.docx]

**SUPPLEMENTARY DOCUMENT 2**

**Detailed information of antibiotic prescription proportion by ICD-10 acute respiratory infection diagnosis**

| **ICD-10 acute respiratory infection diagnosis** | **Number of visits (%) prescribed any antibiotic** | **Number of visits (%) prescribed access-Abs** | **Number of visits (%) prescribed watch-Abs** | **Number of visits (%) prescribed non-recommended-Abs** |
| --- | --- | --- | --- | --- |
| Acute otitis media (n=1962) | 1962 (100) | 1891 (96.4) | 64 (3.3) | 7 (0.4) |
| Acute tonsillitis (n=6721) | 6673 (93.3) | 5885 (87.6) | 723 (10.8) | 65 (1.0) |
| Acute sinusitis (n=3046) | 3022 (99.2) | 2786 (91.5) | 207 (6.8) | 29 (1.0) |
| Acute laryngitis and tracheitis (n=959) | 950 (99.1) | 862 (89.9) | 71 (7.4) | 17 (1.8) |
| Upper ARI of multiple and unspecified sites (n = 3436) | 3400 (99.0) | 3196 (93.0) | 198 (5.8) | 6 (0.2) |
| Pneumonia (n=4524) | 4472 (98.9) | 3921 (86.7) | 511 (11.3) | 40 (0.9) |
| Unspecified acute lower respiratory infection (n=79) | 77 (97.5) | 74 (93.7) | 3 (3.8) | 0 (0.0) |
| Acute bronchitis (n=35619) | 34675 (97.3) | 32397 (91.0) | 2108 (5.9) | 170 (0.5) |
| Acute pharyngitis (n=127322) | 123754 (97.2) | 114858 (90.2) | 6318 (5.0) | 2578 (2.1) |
| Acute nasopharyngitis [common cold] (n=7091) | 6418 (90.5) | 5524 (77.9) | 403 (5.7) | 491 (7.7) |
| Influenza (n=2251) | 1741 (77.3) | 1582 (70.3) | 159 (7.1) | 0 (0.0) |
| Total visits for ARIs (n=193010) | 187144 (97.0) | 172976 (92.5) | 10765 (5.6) | 3366 (1.8) |
