# Supplementary material for: Outpatient antibiotic prescribing for acute respiratory infections in Vietnamese primary care settings by the WHO AWaRe (Access, Watch and Reserve) classification: An analysis using routinely collected electronic prescription data
Source: Lancet Reg Health West Pac. 2022 Oct 11;30:100611. doi: 10.1016/j.lanwpc.2022.100611 (PMC9677071; doi:10.1016/j.lanwpc.2022.100611)
Supplement: Supplementary file 4 [file mmc4.docx]

**Supplementary document 4**

**Table X. Factors associated with watch-antibiotic prescription for adult patients with ARIs**

|  | **Risk ratio** | **95% confidence interval** |
| --- | --- | --- |
| **Gender**  Male  Femal | 1 [reference]  1.00 | -  (0.95-1.05) |
| **Age group, years**  18-49  50-64  ≥65 | 1 [reference]  0.99  0.82 | -  (0.92-1.06)  (0.95-1.05) |
| **District**  Truc Ninh  Nam Truc  Nghia Hung  Xuan Truong  Y Yen  Hai Hau | 1 [reference]  0.37  0.60  3.83  3.55  11.11 | -  (0.11-1.30)  (0.20-1.75)  (1.25-10.60)  (1.29-9.09)  (4.72-22.28) |
| **Acute respiratory infection diagnosis**  [H65] Acute otitis media  [J00] Acute nasopharyngitis [common cold]  [J01] Acute sinusitis  [J02] Acute pharyngitis  [J03] Acute tonsillitis  [J04] Acute laryngitis and tracheitis  [J06] Acute upper respiratory infections of multiple/ unspecified sites  [J09-J11] Influenza  [J12-J18] Pneumonia  [J20-J21] Acute bronchitis/bionchiolitis  [J22] Unspecified acute lower respiratory infection | 2.88  0.60  1.95  1 [reference]  2.41  1.40  1.40  1.28  1.33  1.16  0.65 | (2.19-3.74)  (0.51-0.71)  (1.68-2.26)  -  (2.09-2.76)  (1.06-1.86)  (1.03-1.89)  (0.93-1.72)  (1.17-1.51)  (1.08-1.26)  (0.06-5.47) |
| **Cormobid chronic respiratory illness**  No  Yes | 1 [reference]  1.19 | -  (1.03-1.38) |
| **Visit type**  First visit  Follow-up visit | 1 [reference]  1.15 | -  (1.06-1.26) |

**Table Y. Factors associated with watch-antibiotic prescription for child patients with ARIs**

|  | **Risk ratio** | **95% confidence interval** |
| --- | --- | --- |
| **Gender**  Male  Femal | 1 [reference]  0.95 | -  (1.80-2.06) |
| **Age group, y**  <5  5-17 | 1.92  1 [reference] | (1.80-2.06)  - |
| **District**  Truc Ninh  Nam Truc  Nghia Hung  Xuan Truong  Y Yen  Hai Hau | 1 [reference]  2.00  4.15  3.09  1.74  2.37 | -  (0.57-5.90)  (1.51-9.21)  (1.01-7.77)  (0.58-4.73)  (0.88-5.70) |
| **Acute respiratory infection diagnosis**  [H65] Acute otitis media  [J00] Acute nasopharyngitis [common cold]  [J01] Acute sinusitis  [J02] Acute pharyngitis  [J03] Acute tonsillitis  [J04] Acute laryngitis and tracheitis  [J06] Acute upper respiratory infections of multiple/ unspecified sites  [J09-J11] Influenza  [J12-J18] Pneumonia  [J20-J21] Acute bronchitis/bionchiolitis  [J22] Unspecified acute lower respiratory infection | 1.39  0.64  1.18  1 [reference]  1.40  1.24  1.41  0.77  1.33  1.39  1.07 | (0.71-2.54)  (0.54-0.77)  (0.58-2.26)  -  (1.25-1.57)  (0.73-2.01)  (1.16-1.70)  (0.58-1.01)  (1.11-1.60)  (1.26-1.52)  (0.21-4.00) |
| **Cormobid chronic respiratory illness**  No  Yes | 1 [reference]  0.86 | -  (0.58-1.26) |
| **Visit type**  First visit  Follow-up visit | 1 [reference]  1.10 | -  (1.00-1.21) |
