# Supplementary material for: Outpatient antibiotic prescribing for acute respiratory infections in Vietnamese primary care settings by the WHO AWaRe (Access, Watch and Reserve) classification: An analysis using routinely collected electronic prescription data
Source: Lancet Reg Health West Pac. 2022 Oct 11;30:100611. doi: 10.1016/j.lanwpc.2022.100611 (PMC9677071; doi:10.1016/j.lanwpc.2022.100611)
Supplement: Supplementary file 5 [file mmc5.docx]

**Supplementary Table 4 – List of antibiotics prescribed and proportions of prescribing**

|  | Antibiotic name | Administration route | Pharmacological class (4^th^ level WHO-ATC class) | WHO-AWaRe class | Number of visits prescribed | Proportion of prescribing among studied population |
| --- | --- | --- | --- | --- | --- | --- |
| 1 | Amoxicillin | Oral | Penicillins with extended spectrum | Access | 52,766 | 27.3% |
| 2 | Phenoxymethylpenicillin | Oral | Beta-lactamase sensitive penicillins | Access | 3,907 | 2.0% |
| 3 | Amoxicillin and clavulanic acid | Oral | Combinations of penicillins, incl. beta-lactamase inhibitors | Access | 4,275 | 2.2% |
| 4 | Sulfamethoxazole and trimethoprim | Oral | Combinations of sulfonamides and trimethoprim, incl. derivatives | Access | 16,319 | 8.5% |
| 5 | Cephalexin | Oral | First-generation cephalosporins | Access | 89,939 | 46.6% |
| 6 | Cefadroxil | Oral | First-generation cephalosporins | Access | 4,367 | 2.3% |
| 7 | Cefradine | Oral | First-generation cephalosporins | Access | 16,776 | 8.7% |
| 8 | Cefaclor | Oral | Second-generation cephalosporins | Watch | 811 | 0.4% |
| 9 | Cefuroxime | Oral | Second-generation cephalosporins | Watch | 6,426 | 3.3% |
| 10 | Ciprofloxacin | Oral | Fluoroquinolones | Watch | 1070 | 0.6% |
| 11 | Erythromycin | Oral | Macrolides | Watch | 2,458 | 1.3% |
| 12 | Amoxicillin and cloxacillin | Oral | Combinations of penicillins, incl. beta-lactamase inhibitors | Not-recommended | 3,404 | 1.8% |
